# 

**Published:** 1880-04-20

**Authors:** 


### TABLE OF CONTENTS.

ORIGINAL AND SELECTED ARTICLES.

The Therapeutic Action of Quinine, by J. W. Compton, M.D., of Indiana, 113. Cure of Meniere's Disease, by J. T. Jenkins, M.D., of Kentucky; Etiology of Typhoid Fever, 118. Intra-uterine Medication, by C. D. Palmer, M.D., Cincinnati, Ohio, 120. Litholapaxy, by A. F. Sanger, M.D., Bangor, 124. Traumatic Tetanus, by Dr. H. Van Buren, Chicago, 126. Trismus Neonatorum, 128. Supra-Orbital Neuralgia Cured by Nerve-stretching, 129.

ABSTRACTS AND GLEANINGS.

Treatment of Yellow Fever, 130. Naso-Pharyngeal Catarrh, 131. Surgical Treatment of Pleuritic Effusions, 132. Mysophobia. 133 Hydrastis Canadensis, 135. Wind Spasm, 136. Wickersheimer's Preservative Fluid, 137. Death from Chloroform; Citrate of Caffeine in Asthma, 138. Hemorrhage in Abortion, 139. How to postpone the Use of Spectacles; Treatment of Hepatic Calculi, 140. Apparatus for Fractures of the Clavicle; Coptis Trifoliata—Gold Thread, 141. Counter-Irritation in Febrile Phthisis; Petroleum in Chest Diseases; Glycerine in Phthisis; Phosphorus and its Combinations, 142.

SCIENTIFIC ITEMS.

Paper Pulp from Poplar Wood; Ophthalmoscopic Discovery; Valuable Sanitary Inventions, 143. Silver Oxide; Atoms; Tides in Mines, 144.

PRACTICAL NOTES AND FORMULÆ.

Excipients; Chloroform Vapor in Earache, 145. Guaiacum; For Diphtheria; Dr. Mussey's Prescription for Dysmenorrhœa; Iodide of Potassium and Calomel, 146. Varicella and Vaccination; Damiana; Injury of Eyes from Chloral; Iodoform and Quinia in Ulcers; Cure of Gleet; Hog-Cholera Medicine, 147.

EDITORIAL AND MISCELLANEOUS.

Editorial Notices, 148. The American Medical Association, 149. Our Trip to Cincinnati, 150. Book Notices, 151. Receipted; Special Notices, 152.

